# Functional Neuromuscular Junctions Formed by Embryonic Stem Cell-Derived Motor Neurons

**DOI:** 10.1371/journal.pone.0036049

**Published:** 2012-05-04

**Authors:** Joy A. Umbach, Katrina L. Adams, Cameron B. Gundersen, Bennett G. Novitch

**Affiliations:** 1 Department of Molecular and Medical Pharmacology, David Geffen School of Medicine, University of California Los Angeles, Los Angeles, California, United States of America; 2 Department of Neurobiology, Eli and Edythe Broad Center for Regenerative Medicine and Stem Cell Research, David Geffen School of Medicine, University of California Los Angeles, Los Angeles, California, United States of America; 3 Molecular Biology Interdisciplinary Graduate Program, University of California Los Angeles, Los Angeles, California, United States of America; Stanford University School of Medicine, United States of America

## Abstract

A key objective of stem cell biology is to create physiologically relevant cells suitable for modeling disease pathologies in vitro. Much progress towards this goal has been made in the area of motor neuron (MN) disease through the development of methods to direct spinal MN formation from both embryonic and induced pluripotent stem cells. Previous studies have characterized these neurons with respect to their molecular and intrinsic functional properties. However, the synaptic activity of stem cell-derived MNs remains less well defined. In this study, we report the development of low-density co-culture conditions that encourage the formation of active neuromuscular synapses between stem cell-derived MNs and muscle cells in vitro. Fluorescence microscopy reveals the expression of numerous synaptic proteins at these contacts, while dual patch clamp recording detects both spontaneous and multi-quantal evoked synaptic responses similar to those observed in vivo. Together, these findings demonstrate that stem cell-derived MNs innervate muscle cells in a functionally relevant manner. This dual recording approach further offers a sensitive and quantitative assay platform to probe disorders of synaptic dysfunction associated with MN disease.

## Introduction

All motor functions from locomotion to respiration depend on the communication between motor neurons (MNs) in the spinal cord and muscle cells in different regions of the body. This vital activity is susceptible to many neurodegenerative diseases, most notably amyotrophic lateral sclerosis (ALS) and spinal muscular atrophy (SMA), resulting in MN dysfunction and ultimately death [1], [2]. While progress has been made in identifying genes associated with MN degeneration [3]–[5], the molecular and cellular processes underlying disease onset and progression remain unclear.

Over the past decade, considerable attention has been focused on using stem cell-derived MNs to model disease pathogenesis, driven by demonstrations that mouse and human embryonic stem cells (mESCs and hESCs) can be directed to form MNs in response to developmental signals that promote MN formation in vivo [6]–[9]. Recent studies have further shown that MNs can be similarly produced from induced pluripotent stem cells (IPSC) including those derived from ALS and SMA patients [10]–[12], and through transcription factor-mediated reprogramming of fibroblasts [13]. A remaining challenge, however, is to establish methods to evaluate the function of normal and diseased MNs obtained from these sources in a physiologically relevant setting.

An important step towards this goal is the development of in vitro assays to measure the synaptic activity of MNs at neuromuscular junctions, as many studies have pointed to synaptic dysfunction as an early readout and possibly an initiating event in MN disease progression [14], [15]. ESC and IPSC-derived MNs have previously been shown to exhibit many molecular and physiological properties associated with mature MNs [12], [16], [17]. Moreover, when transplanted into the embryonic chick spinal cord [9], [18], [19] or peripheral nerve of mice [20], these neurons appear to be capable of extending axons towards peripheral muscle targets. Despite these successes, relatively little attention has been placed on direct measurements of the communication between stem cell-derived MNs and muscle cells. In part, this reflects the inherent difficulties in isolating connected pairs of cells in mass culture or transplantation settings.

In this study, we report the development of low-density culture conditions that encourage the formation of neuromuscular junctions between isolated ESC-derived MNs and muscle cells. This system enables the direct measurement of synaptic communication through dual patch clamp recordings. In this setting, MNs form neuromuscular junctions containing functionally importan synaptic proteins, and these synapses exhibit both spontaneous and stimulus-evoked transmitter release. Together, these findings constitute an important advance in validating the functional identity of stem cell-derived MNs and providing a platform for defining their synaptic properties under normal and diseased conditions.

## Results

### ESC-derived MNs form cholinergic synapses on muscle cells under low-density co-culture conditions

**Figure 1:**
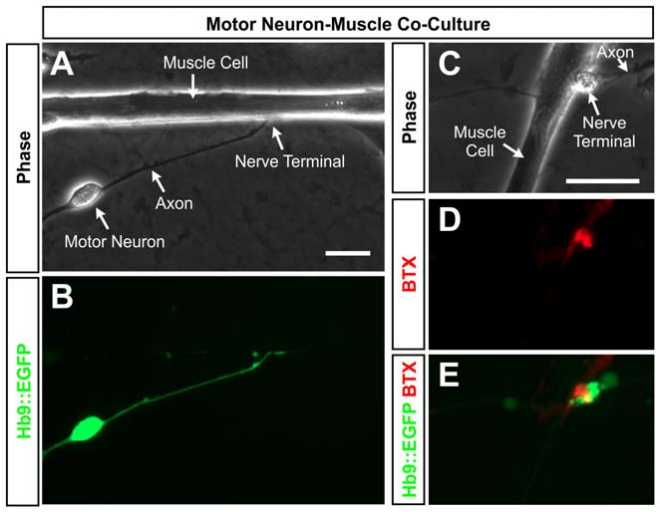
Morphology of neuromuscular junctions formed in vitro by mESC-derived MNs. (A, B) Brightfield and fluorescence images showing an Hb9::EGFP^+^ ESC-derived MN extending an axon to contact a muscle cell under low-density cell culture conditions. (C–E) The adjacent trio of images shows a representative axonal varicosity contacting a muscle cell stained for nicotinic ACh receptors using fluorescent α-bungarotoxin (BTX) overlaid with Hb9::EGFP fluorescence in the MN terminal bouton. Scale bars are 20 µm.

**Figure 2:**
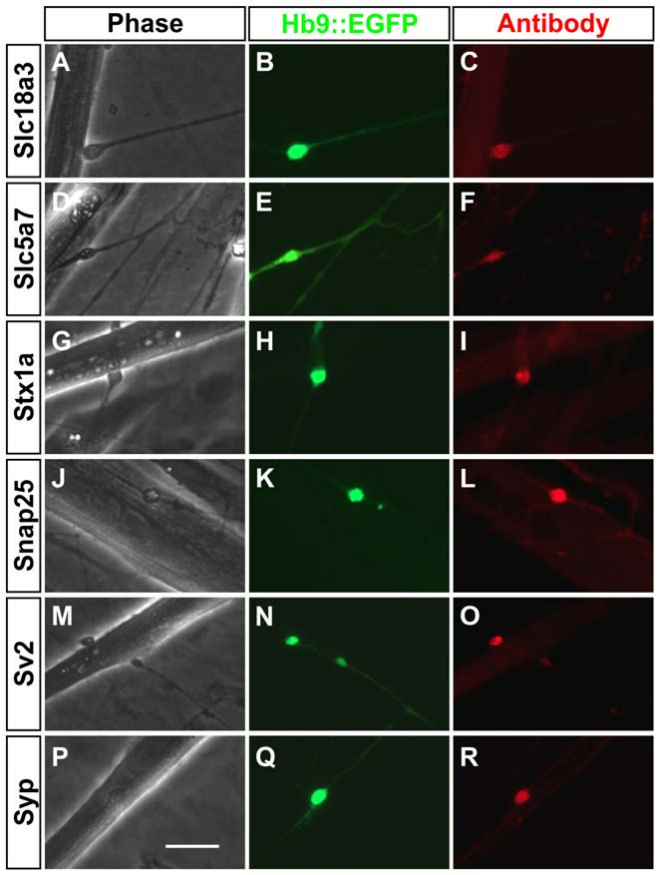
mESC-derived MNs form cholinergic synapses with muscle cells in vitro. Immunofluorescence analysis of proteins expressed at nerve terminals of mESC-derived MNs. The three columns show: (A, D, G, J, M, P) brightfield images of axon terminals contacting muscle cells; (B, E, H, K, N, Q) green fluorescence associated with the Hb9::EGFP MN reporter; (C, F, I, L, O, R) red fluorescence corresponding to antibody staining for the indicated presynaptic proteins: Slc18a3 (VAChT), Slc5a7 (ChT1), Syntaxin 1a (Stx1a), Snap25, Sv2, and Synaptophysin (Syp). Scale bar is 20 µm.

To evaluate the synaptic activity of ESC-derived MNs, we first developed culture conditions that were amenable to patch clamp analysis of MN-muscle pairs. The initial step was to test whether cells could form synaptic contacts when plated at low density (1.2×10^4^ muscle cells and ∼1.2×10^4^ Hb9::EGFP^+^ MNs per 35 mm dish). We reasoned that such conditions might encourage the preferential growth of motor axons to nearby partners and minimize non-synaptic contacts made when cells are plated at high densities. Under these conditions, each culture dish yielded 1–4 isolated MN-muscle cell pairs with Hb9::EGFP^+^ axons projecting towards spindle-shaped muscle cells (Fig. 1A, B). At the point of contact between the axons and muscle cells there was a varicose enlargement of the terminal bouton (Figs. 1 and 2). Bouton diameter ranged from 3–11 µm in diameter with a mean diameter of 6.9±2.0 µm (n = 65) and was easily distinguished from motor neuron soma, which were typically >20 µm in diameter. This geometry of neuron-muscle pairing was sufficiently common that it enabled the reliable identification of nerve and muscle cells that were likely to have made functional synaptic contacts. The presence of α-bungarotoxin (BTX) staining (Fig. 1C–E) further indicated that nicotinic ACh receptors preferentially accumulated at these sites.

We next used immunofluorescence microscopy to investigate whether other macromolecules characteristic of cholinergic synapses were present at the nerve-muscle contacts. Proteins associated with ACh metabolism including Slc18a3 (VAChT) and the high affinity choline transporter Slc5a7 were detected at these sites along with the SNARE proteins Snap25 and Syntaxin 1a (Fig. 2A–L). Concomitantly, synaptic vesicle proteins such as synaptophysin and SV2 were also present (Fig. 2M–R). These results were representative of data obtained from at least two separate culture dishes for each antibody. In each dish, at least 4 neuromuscular junctions were imaged and every Hb9::EGFP^+^ terminal showed immunoreactivity for these presynaptic proteins. Collectively, these data indicate that sites of contact between ESC-derived MNs and muscle cells contain components of the molecular machinery associated with cholinergic synapses.

### Neuromuscular synapses formed in vitro are functional and trigger both spontaneous and evoked muscle contractions

**Figure 3:**
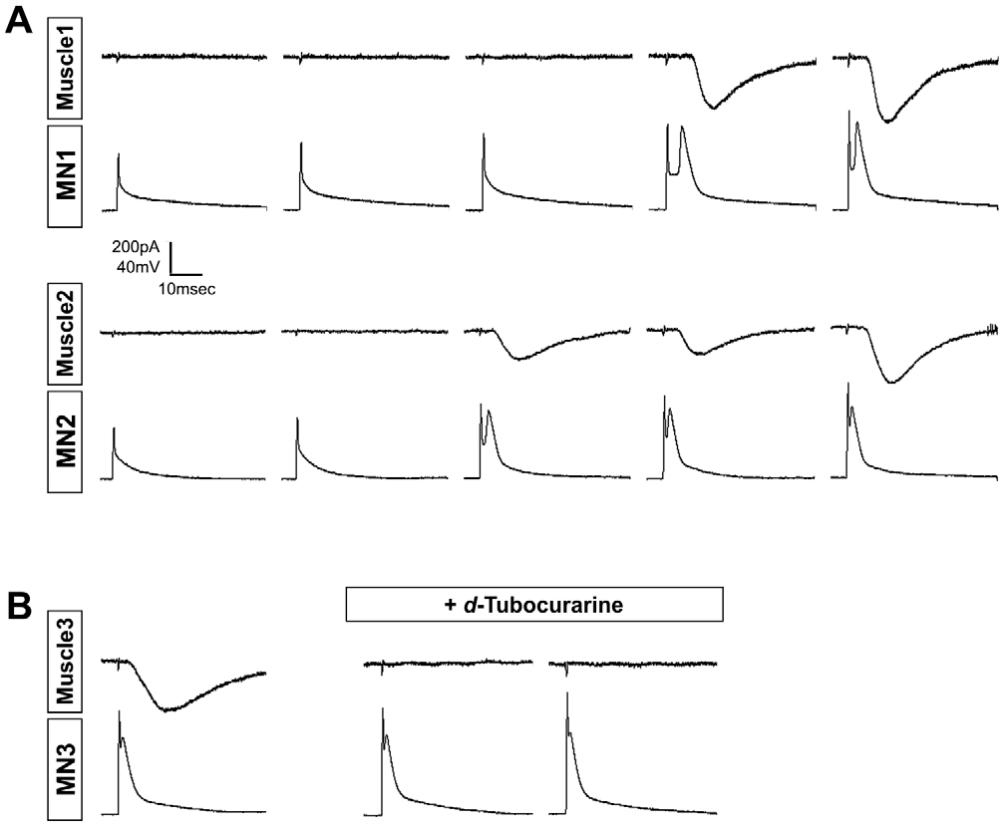
mESC-derived MNs trigger a post-synaptic response in muscle cells only when MNs fire action potentials. (A) In each pair of recordings, the lower trace shows the MN response to depolarizing currents of increasing amplitude. For both MN1 and MN2, current was increased in 0.5 nA steps from 2.5 nA on the left to 4.5 nA on the right. Stimuli were delivered at 30 s intervals. MN responses were initially passive, but upon reaching threshold, MNs typically fired a single action potential with this stimulus duration. Each MN action potential elicited an EPC of variable amplitude after a delay of 1–3 msec. (B) Addition of *d*-tubocurarine (10 µM) eliminated the stimulus-evoked EPC, but did not affect the MN action potential.

To determine whether the nerve-muscle contacts formed in culture exhibit the functional properties of neuromuscular junctions, we sealed patch clamp pipettes onto MN-muscle pairs. Current injection into the ESC-derived MNs initially elicited passive membrane responses in the MNs, but no electrical response in muscle cells beyond the stimulus artifact (Fig. 3A). However, once threshold was exceeded, the MNs fired an action potential. With a brief delay (1–3 msec), MN action potentials were followed by an excitatory post-synaptic current (EPC) in the muscle cells (Fig. 3A). These EPCs are reminiscent of the classical electrophysiological signature of synaptic communication between MNs and muscle cells observed in en-bloc preparations [21].

**Figure 4:**
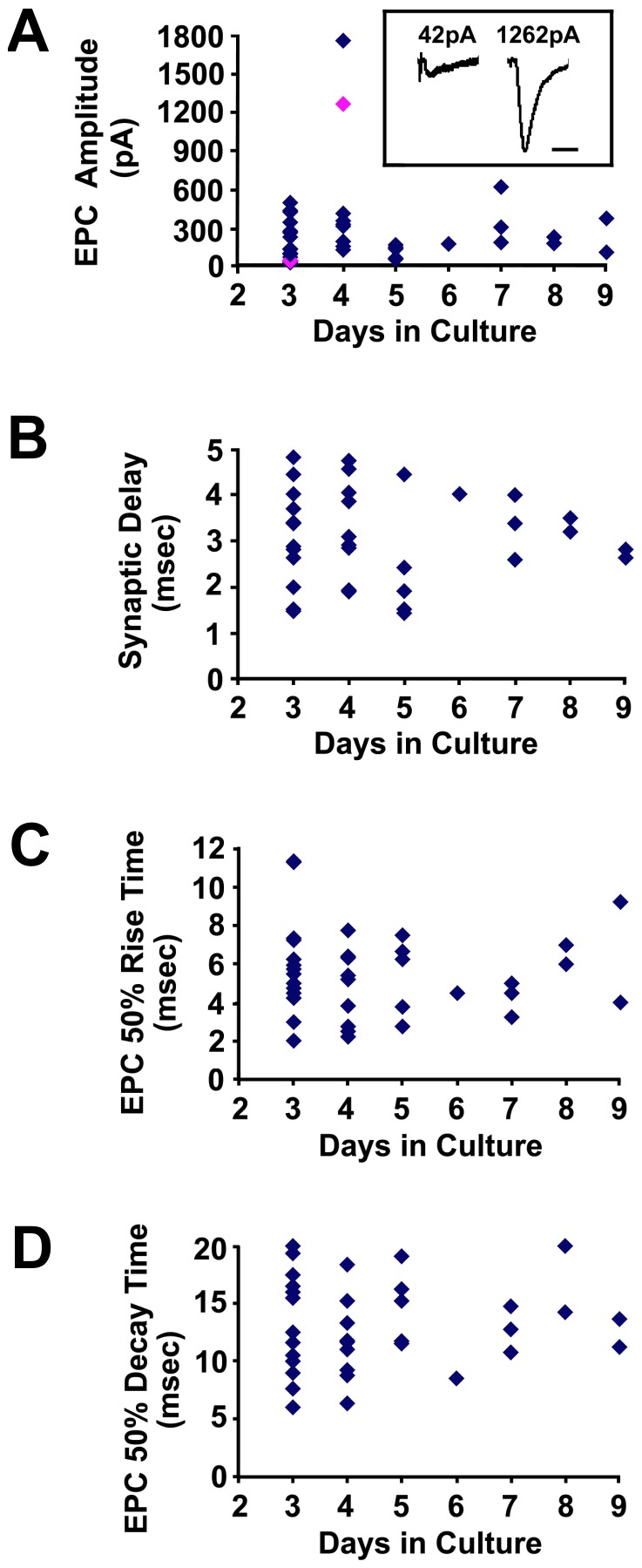
Quantification of the properties of mESC-derived MN-muscle synapses. (A–D) Scatter plots show the variability of the following parameters as a function of days in co-culture: (A) EPC amplitudes, where each point is the largest EPC recorded at each synapse. Inset shows representative EPCs that correspond to the fuchsia diamonds (time scale is 25 msec); (B) synaptic delay, measured from the peak of the MN action potential to the start of the EPC; (C) EPC rise time; (D) EPC decay time.

From 60 dishes examined, 111 neuron-muscle pairs with geometries similar to that shown in Fig. 1A were identified. Successful patches (where the resting potential in both cells was >−30 mV) were obtained for ∼37% of these pairs (41/111), of which ∼90% (37/41) showed functional synaptic responses as illustrated in Fig. 3A. Overall, muscle resting membrane potentials averaged −53.6±9.5 mV; S.D., while neuron resting potentials were 40.9 mV±9.4 mV; S.D. The records in Fig. 3A also illustrate the variability in the synaptic delay, amplitude and time course of EPCs. Scatter plots summarize the observed range of synaptic delays, EPC amplitudes, EPC rise times, and EPC decays (Fig. 4A–D). In particular, EPC amplitude varied from <100 pA at some synapses on day 3 to >1 nA on 4 day (Fig. 4A). EPCs were abolished by the addition of the nicotinic ACh receptor antagonist, *d*-tubocurarine (10 µM, n = 3 trials; Fig. 3B), confirming the cholinergic nature of these EPCs. EPCs were similarly eliminated by replacement of extracellular Ca^2+^ with 10 mM Mg^2+^ (n = 3 trials; data not shown). Moreover, in parallel experiments using a patch pipette on the neuron alone, triggering of neuronal action potentials led to visible muscle contractions in ∼20% of the trials evaluated (3/15; data not shown). Collectively, these data indicate that the neuromuscular junctions formed in these cultures exhibit stimulus-evoked and Ca^2+^-dependent neurotransmitter release capable of triggering muscle contraction.

**Figure 5:**
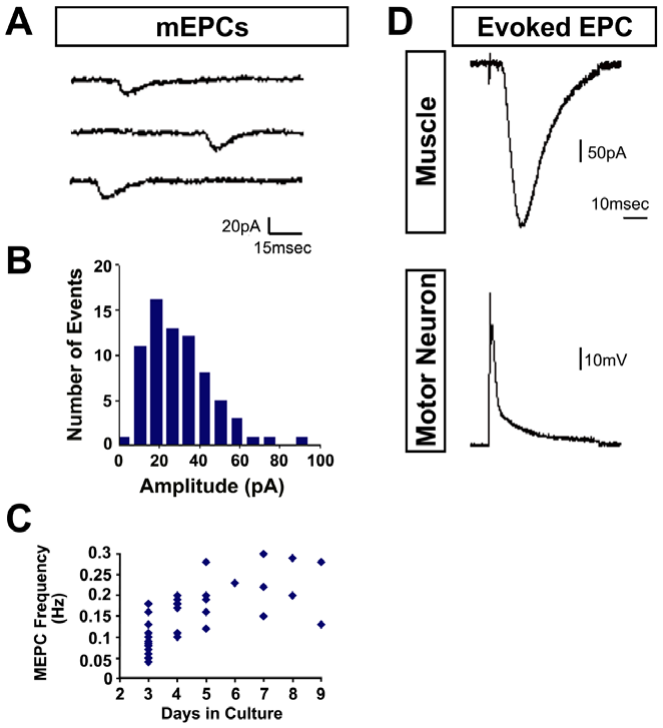
mESC-derived MNs exhibit both spontaneous and evoked synaptic currents at neuromuscular junctions formed in vitro. Patch pipettes were sealed onto MNs and muscle cells making contact as shown in Fig. 1. (A, B) Representative records of spontaneous mEPCs in a muscle cell and their amplitude distribution. (C) Scatter plot of mEPC frequency over different times in culture. (D) Current injection into the MN triggers an action potential that elicits a multi-quantal EPC in the muscle cell.

An important advantage of the dual patch configuration is that it enables one to conduct additional quantitative analyses of both spontaneous and evoked synaptic events at these neuromuscular junctions. We thus evaluated the profile of spontaneous miniature (m) EPCs recorded in muscle cells for which evoked EPCs were also obtained (Fig. 5A–D). The mEPC amplitude distribution (Fig. 5B) is typical of the skewed Gaussian distribution observed at 70% of the nerve-muscle contacts in these cultures. Cumulatively, mEPC frequencies ranged from 0.04–0.30 Hz, with mEPC frequencies <0.1 Hz characteristic of 3 d old cultures and >0.1 Hz after 4 days in culture (Fig. 5C). Although we have not yet undertaken a systematic evaluation of the quantal content of the EPCs in this system, it is important to note that EPCs such as that shown in Fig. 5A are very likely comprised of multiple quanta. This conclusion derives from the fact that the amplitude of this EPC is at least five times greater than the largest mEPC (Fig. 5A, B, D). Based on this criterion, multi-quantal EPCs were observed in ∼95% (36/37) of the neuromuscular junctions from which mEPC/EPC recordings were obtained. Taken together, these data indicate that mESC-derived MNs are capable of coupling action potentials to the synchronous release of multiple quanta at these nerve-muscle contacts to elicit muscle contractions.

## Discussion

The definitive feature of MNs is their ability to form functional neuromuscular junctions and thereby drive the contraction of skeletal muscle cells. Our study provides critical evidence that ESC-derived MNs can exhibit robust synaptic communication with muscle cells under simplified in vitro culture conditions. Empirically, this is a significant observation, as the use of stem cell-derived MNs for regenerative purposes or disease modeling requires that the cells faithfully mimic their natural counterparts in both molecular and functional properties. Our data show that ESC-derived MNs express several proteins, including nicotinic ACh receptors, Slc18a3 (VAChT), the high affinity choline transporter Slc5a7, and SNARE proteins found at native neuromuscular junctions, and exhibit both spontaneous and action potential-dependent, multi-quantal secretion of ACh to trigger post-synaptic potentials and muscle contraction. These results further provide an important extension to previous studies that have used bath application of glutamate to evoke post-synaptic potentials and muscle contraction in high-density MN-muscle cell cultures [13], [16], [22]. Moreover, the ability to quantify the functional properties of individual nerve-muscle contacts offers the opportunity to rigorously assess the impact of a variety of experimental manipulations on these synaptic events.

To define the synaptic activity of MN-muscle pairs, our investigation was intentionally restricted to the differentiated progeny of mESCs and C2C12 muscle cells. Our data indicate that MN-muscle synapses formed under these simplified conditions recapitulate many features of neuromuscular communication seen in vivo. For example, the frequency of spontaneous mEPCs (0.04–0.3 Hz) is within the range reported for muscle fibers of late embryonic and early post-natal rodents [23]–[26]. Similarly, the rise and decay times of EPCs are within the range observed for developing rat neuromuscular junctions [23]. It is important to note that functional MN-muscle synapses formed under these conditions typically survived for a maximum of 8–9 days in culture. This limitation might reflect a lack of support provided in vivo by other cells including astrocytes and Schwann cells or presynaptic inputs to the MNs from spinal interneurons. The low-density culture conditions established in this study provide a suitable platform for evaluating the influence of different cell types in future work. Nevertheless, we have found that similar co-culture of human ESC and IPSC-derived motor neurons with muscle cells results in synaptic contacts that persist for several weeks (JAU, KLA, and BGN, unpublished data), suggesting that at least some aspects of synaptic stability are inherent to the MNs themselves and highly variable between species.

During embryonic development, different classes of MNs exhibit a high degree of selectivity in their choice of muscle targets [27], [28]. However, we infer from the present results that the programs that dictate motor innervation patterns are sufficiently malleable such that ESC-derived MNs can form functional synapses on C2C12 cells. Although this observation is not surprising given the promiscuity of mammalian MNs for forming neuromuscular junctions in vitro [29], precise matching of MN and muscle subtypes might nevertheless be crucial for ensuring full synaptic activity and stability [30]. Progress has recently been made in understanding the mechanisms underlying MN fate selection [27], [31], [32], and it should be fruitful to determine whether this information can be harnessed to bias the differentiation of mESC-derived MNs to favor the innervation of specific classes of muscle cells both in vitro and in vivo.

Another important use for this co-culture system will be for modeling neuromuscular disorders. There is abundant evidence for an early and profound impairment of neuromuscular transmission in amyotrophic lateral sclerosis [33], and we showed previously that mutant forms of superoxide dismutase 1 (SOD1) alter the morphology and survival of hESC-derived MNs in vitro [34]. Consequently, conditional expression of mutant SOD1 in MN-muscle co-cultures is likely to provide an informative system for clarifying the impact of SOD1 mutant alleles on nerve-muscle communication. Similarly, recent data suggest that proprioceptive circuits may be particularly vulnerable in spinal muscular atrophy [35]. The in vitro system developed here might accordingly be expanded to assess the underlying cellular and molecular mechanisms that contribute to this decline in synaptic input to MNs. Thus, in addition to their utility for helping to answer fundamental biological questions, these co-cultures have clear applications in addressing problems of medical significance.

## Materials and Methods

### Differentiation of mESCs

Hb9::EGFP mESCs [9] were maintained and differentiated into MNs as previously described [9], [36]. Briefly, mESCs were plated on 60 mm bacterial petri dishes in core MN medium to elicit embryoid body (EB) formation. Core MN medium consisted of a 1∶1 mixture of Dulbecco's Modified Eagle's Medium/F12 (DMEM/F12) and Neurobasal Medium supplemented with Knockout Serum Replacement, Glutamax, and 2-mercaptoethanol (560 nM), Penicillin/Streptomycin, and Primocin (50 µg/ml; Invivogen). Except as noted, media components were obtained from Invitrogen. After 1 d in culture, EBs were pipetted through a 100 µm strainer to remove large aggregates. The next day, EB culture media was replaced with MN differentiation medium [core MN medium containing N2 supplement (1×), Retinoic Acid (1 µM; Sigma) and Purmorphamine (1.5 µM; EMD Biosciences)]. After 5 d of differentiation, EBs were dissociated using papain (0.5 U/ml; Worthington) in HBSS for 20 min at 37°C with gentle trituration. Cells were collected by centrifugation and washed with MN differentiation medium prior to plating with muscle cells.

### Co-culture of MNs and C2C12 muscle cells

C2C12 cells (CRL-1772) were obtained from the American Type Culture Collection and cultured in myoblast growth medium [DMEM supplemented with 15% fetal bovine serum (FBS), L-glutamine (1 mM) and antibiotics as above]. When the cells reached 60–70% confluence, they were washed with PBS and transferred to muscle differentiation medium [DMEM with 0.5% FBS, insulin (10 µg/ml)-transferrin (5.5 µg/ml)-selenium (39 nM), L-glutamine (1 mM) and antibiotics]. After 2 d, the medium was supplemented with cytosine arabinoside (Ara-C; 10 µM), and cells were cultured for another 2 d to eliminate dividing cells. Differentiated myotube cultures were dissociated using trypsin (0.05%) and plated at low density on Matrigel-coated 35 mm culture dishes (1.2×10^4^ cells/dish) in differentiation medium containing 1 µM Ara-C. 1–2 d after muscle cells were plated, 1.2×10^5^ mESC-derived cells, of which at least 10% were Hb9::GFP^+^ MNs, were added to each dish and the medium was changed to core MN medium supplemented with Brain-Derived Neurotrophic factor (10 ng/ml), Glia-Derived Neurotrophic Factor (10 ng/ml) and Ciliary Neurotrophic Factor (20 ng/ml); neurotrophic factors were obtained from Prospec. Within 1–2 d, motor axons made contact with muscle cells, and functional nerve-muscle contacts were observed for at least 6–7 d.

### Fluorescence microscopy and immunostaining

Cultures were fixed in 3% paraformaldehyde in PBS for 15 min, washed twice with PBS, permeabilized with 0.1% Triton X-100 in PBS for 15 min, and blocked in 10% normal goat serum in PBS for 15 min. Primary antibodies (0.5–2 µg/ml) were added and specimens were incubated for 12–16 h at 4°C. After extensive PBS washes specimens were incubated with secondary antibodies (Alexa 594 goat anti-mouse or goat anti-rabbit IgG; Invitrogen) for 1 h followed by PBS washes. The following primary antibodies were used: Slc18a3 (VAChT, Millipore; AB1588); Slc5a7 (choline transporter 1 (ChT1), Millipore; AB5966)); Sv2 (Developmental Studies Hybridoma Bank); Snap25 (Stressgen; VAP-SV0002); synaptophysin (Syp, Sigma; S-5768); syntaxin 1a (Stx1a, Stressgen; VAM-SV013). Nicotinic acetylcholine (ACh) receptors were detected through bath application of Alexa 594-α-bungarotoxin (Invitrogen) to the cultures prior to fixation. Epifluorescence images were obtained using an Olympus IX70 inverted microscope equipped with a Sensicam cooled CCD camera (PCO) and a Lambda 10 shutter (Sutter Instruments) controlled by Axon Instruments Imaging Workbench. Images were processed using Adobe Photoshop and CorelDRAW software.

### Electrophysiology

Cultures were screened for isolated MN-muscle cell pairs where the axon branched minimally and the axon terminal formed a visible contact with a muscle cell that was <0.1 mm from the cell body. Patch pipettes were sealed sequentially onto both cells using methods described in [37]. Pipette solutions were in mM, muscle: K-gluconate (140), Hepes (10), CaCl_2_ (1), MgCl_2_ (1), EGTA (11), QX-314 (5); neuron: K-gluconate (140), Hepes (10), EGTA (1), Mg-ATP (4), Na-GTP (0.3). The bath solution for recording was in mM: NaCl (120), KCl (1.9), KH_2_PO_4_ (1.2), Na-bicarbonate (20), CaCl_2_ (2.2), MgCl_2_ (1.4), Hepes (7.5). In all cases, pH was adjusted to 7.2. Cells with resting potentials <−30 mV were discarded. After an initial period to record at least 25 spontaneous miniature excitatory post synaptic currents (mEPCs) in the muscle cell (voltage clamped at −80 mV), MNs (maintained in current clamp mode at approximately −70 mV) were stimulated by 0.5 msec current injections of increasing amplitude from +0.5 to +6 nA. Data were collected using Axopatch 2B patch clamp amplifiers with 4-pole Bessel filtering at 5 kHz. Signals were digitized and stored using pClamp and Axotape software (Axon Instruments) and analyzed using pClamp and miniAnalysis (Synaptosoft).
